# Epithelial Ovarian Cancer With Cardiac Metastases in Pregnancy: A Case Report

**DOI:** 10.7759/cureus.107192

**Published:** 2026-04-16

**Authors:** Tatiana Quijano, Luis Puentes, Edmundo Mora

**Affiliations:** 1 Gynecologic Oncology, Ciudad de la Salud Seguro Social Panama, Panama, PAN; 2 Gynecologic Oncology, Universidad Javeriana, Bogotá, COL; 3 Gynecologic Oncology, Fundación Universitaria de Ciencias de la Salud, Bogotá, COL

**Keywords:** carcinoma ovarian epithelial, cardiac metastasis, epithelial ovarian cancer, epithelial ovarian carcinoma, metastasis, ovarian mucinous adenocarcinoma, pregnancy

## Abstract

A 22-year-old primigravid patient in the 18th week of pregnancy presented with epithelial ovarian cancer. At 20 weeks, surgery was performed, and the first cycle of chemotherapy was administered. At 32 weeks, a segmentary cesarean section and oncological surgery were performed. The patient died in late postpartum, and multiple organ failure and multiple metastases were reported in her autopsy. This case shows the importance of evaluating pregnant patients with cancer in coordination with a multidisciplinary team.

## Introduction

Adnexal masses occur in 1/600 to 1/1500 pregnancies, of which 1%-3% are malignant, and these malignancies are usually non-epithelial [1]. Malignant germ cell tumors are the most common ovarian cancer in pregnancy, most often taking the form of dysgerminomas, which represent 38% of cases [2]. The treatment for ovarian carcinoma consists of surgery and chemotherapy, even during pregnancy. The survival rates depend on the stage of the disease and are low in the advanced stages. The most common sites of metastasis are, in order of decreasing frequency, the liver, lymph nodes, lungs, bone, and brain.

Cardiac metastasis is extremely rare: only one case has been reported. Cardiac metastases result from spread by continuity, dissemination, or lymphatic permeability. These metastases commonly present as pleural mesothelioma, lung cancer, melanoma, or breast cancer [3]. A review of autopsies of patients who died of ovarian cancer found metastatic lesions in the pericardium in 7.2% of cases [4]. The aim of the present study was to present a case of a 22-year-old pregnant patient with epithelial ovarian cancer and cardiac metastasis.

## Case presentation

**Figure 1:**
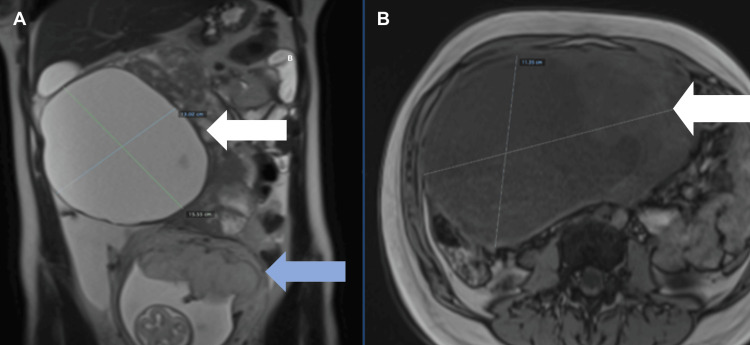
Abdominal-pelvic magnetic resonance imaging without contrast. (A) Coronal and (B) axial images. The white arrow points to the mass, and the blue arrow points to the fetus.

A 22-year-old primigravida in the 18th week of pregnancy presented with low abdominal pain associated with the incidental finding of a complex 13-cm pelvic mass. A physical examination of the patient showed that a fetal heart rate was present, and a fixed, indurated mass of 15 cm was found in the right flank and mesogastrium. The patient was hospitalized. Non-contrast abdominal-pelvic MRI was performed, and the report showed ascites and an expansive abdominal-pelvic mass measuring 17 cm (Figure 1). Tumor markers were elevated (Table 1). A gynecology medical board recommended surgery. The risks of surgery were explained to the patient, who decided to continue the pregnancy.

**Table 1:** Tumor markers at 20 weeks. CEA: carcinoembryonic antigen; U: units; mL: microliters; ng: nanograms.

Tumor markers	Value	Reference range
CA 125	168 U/mL	0-65 U/mL
CA 19-9	2,910 U/mL	0-37 U/mL
CEA	26.4 ng/mL	<3 ng/mL
Alpha-fetoprotein	35.2 ng/mL	10-150 ng/mL

**Figure 2:**
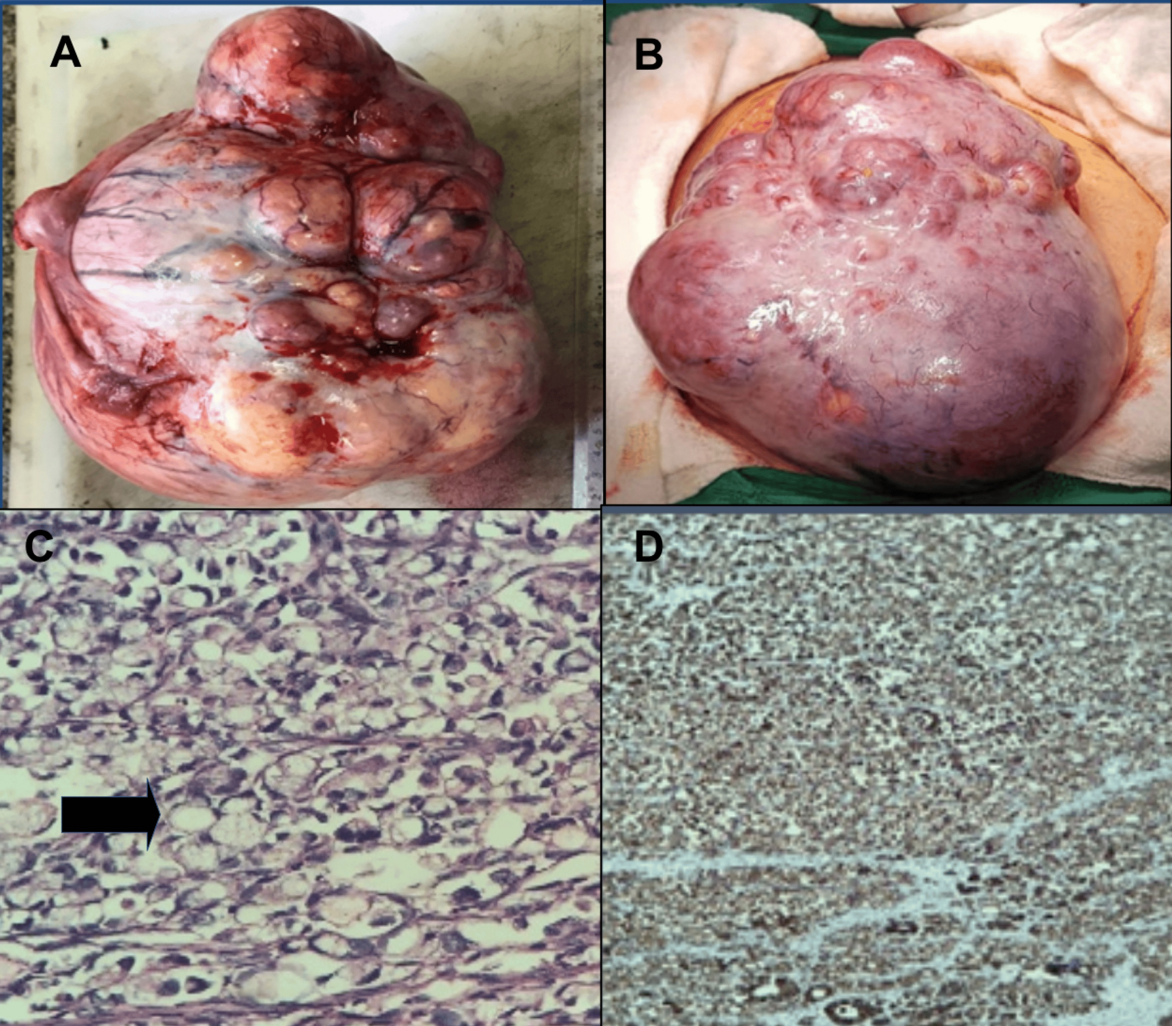
(A-B) Cystic solid mass measuring 16 x 13 x 8 cm, lobed and heterogeneous in appearance, with white and yellow areas, and a solid area measuring 16 x 6 cm weighing 2,406 grams. (C) Staining with hematoxylin and eosin at 100x. The black arrow points to a signet cell ring. (D) Immunohistochemistry PAX8+ at 100x.

At 20 weeks of pregnancy, mass resection and omentectomy were performed, revealing a hypervascularized abdominal pelvic mass of 20 x 18 cm with an irregular surface and intact capsule. The left ovary appeared normal, and no visible lesions were encountered in the liver or the gastric, intestinal, peritoneal, or omental surfaces. Histopathological examination reported a poorly differentiated mucinous ovarian adenocarcinoma. Microscopic implants in the omentum served to determine that the adenocarcinoma was stage IIIa2 (Figure 2).

**Figure 3:**
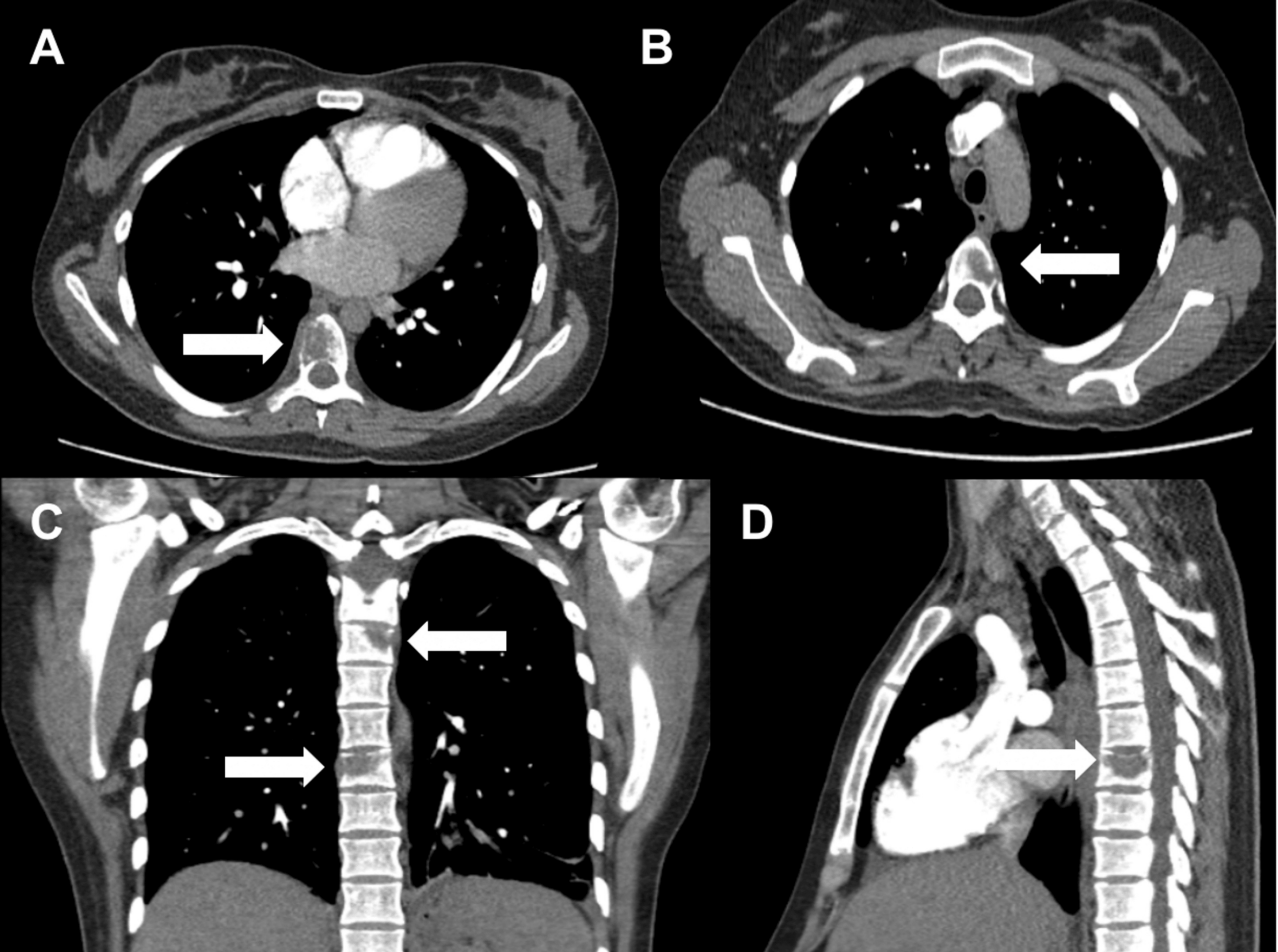
Chest computerized axial tomography with contrast. (A) Axial: T8 level lytic lesion in the vertebral body. (B) T5 axial cut in the vertebral body lytic lesion. (C) Coronal: lytic lesions observed in T5-T8. (D) Sagittal: lytic lesion at the T8 level. The white arrows point to a lytic lesion.

A new medical board decided to start chemotherapy with carboplatin and paclitaxel. The patient was informed about the risks and benefits, and she decided to continue the pregnancy, agreed to the treatment, and gave her informed consent. At 29 weeks, the first cycle of chemotherapy was administered. At 30 weeks’ gestation, the patient presented to the emergency department with severe pain, and fetal well-being was confirmed. Angiotac was performed to rule out pulmonary embolism. The report showed multiple osteolytic lesions. Thoracic computerized axial tomography was then performed, and osteolytic lesions were identified in vertebral bodies C7, T1, T4, T1, and T12 (Figure 3).

Fetal maturation (betamethasone 12 mg c/24 h) was completed. At 32 weeks, three weeks after chemotherapy was completed, a segmentary cesarean section and oncological surgery (hysterectomy, bilateral salpingo-oophorectomy, omentectomy, and appendectomy) were performed. A live male infant weighing 1,645 g was delivered, with Apgar scores of 8, 9, and 9, and no complications or signs of myelosuppression.

Radiation therapy was initiated in late postpartum (17 days) with palliative intention over the cervical and thoracic spine at 300 to 3,000 centigray daily. Eight sessions were completed. The patient died 24 days postpartum. An autopsy reported multiple organ failure, poorly differentiated primary adenocarcinoma of the ovaries, and metastases in the lungs, heart, lymph nodes, and bone marrow.

## Discussion

A pregnant patient presented with mucinous adenocarcinoma, and an autopsy revealed that she had cardiac metastases. The incidence of ovarian cancer during pregnancy is low. Nazer et al. reported that, of 7,785,583 pregnancies between 2003 and 2011, only 19,591 (0.25%) involved a diagnosis with adnexal masses, and only one in 200 of these masses was malignant, resulting in an incidence of 0.12 ovarian cancers per 10,000 pregnancies [1]. Malignant germ cell tumors are the most common type of ovarian cancer in pregnancy, with dysgerminoma being the most frequent subtype, representing 38% of the total [5].

Blake et al. investigated 105 cases of pregnant patients with epithelial ovarian cancer and found that the most common histologies were serous (47.6%) and mucinous (27.6%). Approximately 63.8% of these cancers were diagnosed in the early stages, with 45.3% being diagnosed during the first trimester. In their series, live births were achieved in 81.3% of cases, of which 71.6% were by cesarean section. Nearly all of the patients (99.9%) received surgical treatment, and 55.2% received chemotherapy [2].

According to an analysis of the Surveillance, Epidemiology, and End Results (SEER) program (1973-2015), mucinous epithelial ovarian cancer was the second most common histotype (13.7%) [5]. Mucinous histology typically occurs in young patients. Thus, in the analysis, 26% of the cases were in patients under 44 years of age; similarly, our patient was 22 years old. While mucinous ovarian cancer occurs in the early stages in 65%-80% of patients according to another analysis of the SEER report, the overall three-year survival rate was 91% for localized stages, decreasing to 22.1% for distant disease [6]. In our case, the patient was initially diagnosed at stage IIIa2 because of microscopic disease in the omentum, and, despite the chemotherapy, the disease progressed rapidly.

Tumor markers in pregnancy must be interpreted with caution because physiological changes can influence them [5]. Thus, CA 125 often peaks in the first trimester and normalizes thereafter, and mucinous tumor marker carcinoembryonic antigen (CEA) may be elevated during the third trimester. In our patient, the markers were significantly altered, with a clear bias toward the mucinous origin because of the significant elevation of CEA and CA 19-9 [7].

Ultrasonography remains the initial diagnostic approach of choice. However, any suspicious mass should be evaluated by magnetic resonance imaging (MRI) to determine the mass characteristics, abdominal extension, and lymph involvement. Further, gadolinium crosses the placental barrier, and computed tomography scans expose the fetus to ionizing radiation, which is associated with risk to fetal development and viability and, therefore, is contraindicated during pregnancy [7,8]. In our case, a non-contrast MRI was performed as indicated in the literature. MRI correctly visualized the disease as being localized to the pelvis.

The treatment for ovarian cancer is surgical management, followed by chemotherapy, and should be the same regardless of pregnancy. In the early stages, surgery to preserve fertility is an acceptable oncological option. In the advanced stages, interruption of the pregnancy and surgical management to attempt complete cytoreduction should be considered. If such surgery cannot be done, neoadjuvant chemotherapy is an option [5-8]. The aim of surgical procedures should be to avoid both rupture of the mass and intraperitoneal spillage. While laparoscopy is safe during pregnancy, the surgeon’s skill, size of the mass, gestational age, and the extent of surgery must be carefully weighed. In the resection of our patient's mass, omentectomy, and inspection of the abdominal cavity, the approach involved laparotomy because of the size of the mass.

Platinum and taxane-based regimens are the chemotherapy of choice for epithelial ovarian cancer, including during pregnancy, though these approaches are contraindicated during the first trimester. The use of chemotherapeutics during pregnancy carries significant risks to the fetus, including restriction of intrauterine growth, intrauterine death, premature labor, preterm birth, small for gestational age, and admission to a neonatal intensive care unit [2,5]. Blake et al. reported the incidence of low birth weight to be 26% in pregnant patients who received chemotherapy, compared with 4%-8% in the normal population [2]. To mitigate secondary hematological toxicity, chemotherapy should be discontinued at least three weeks prior to the expected delivery date [8].

In advanced epithelial ovarian cancer, the common metastasis sites include the liver, lymph nodes, lungs, bones, and brain [9]. Cardiac metastases occur through direct extension as a result of continuity, hematogenous spread, and lymphatic permeability. A review of autopsies of patients who died of ovarian cancer found pericardial involvement in 7.2% of cases [4]. Bussani et al. analyzed autopsies and reported finding common cardiac metastasis in pleural mesothelioma, lung cancer, melanoma, and breast cancer, with 9% of ovarian cancer cases having epicardial involvement but none showing myocardial infiltration [3].

To our knowledge, there has been only one other case report in the literature of a patient with myocardial metastases from mucinous ovarian cancer (stage IIb) who was treated with surgery and chemotherapy [10]. In that case, the patient was 64 years old, and recurrence occurred eight months after surgery. The patient died one month later from heart failure, and her autopsy revealed invasive anaplastic carcinoma lesions in the lung parenchyma and myocardium (Table 2). The presence of anaplastic or sarcomatous features confers significantly greater aggressiveness compared with purely mucinous tumors.

**Table 2:** Comparative analysis of cardiac metastasis in ovarian cancer.

Author	Patient	Histology	Stage	Chemotherapy	Surgery	Follow-up
Kihara et al. (2019) [10]	64 years	Ovarian mucinous adenocarcinoma	IIb	Paclitaxel and carboplatin (6 cycles)	Hysterectomy, bilateral salpingo-oophorectomy, omentectomy, appendectomy, and intrapelvic and para-aortic lymph node dissection	Recurrence at 8 months; death 1 month later
Current case (2024)	22 years	Ovarian mucinous adenocarcinoma	IIIa2	Paclitaxel and carboplatin (1 cycle)	Mass resection and omentectomy (initial at 20 weeks). Hysterectomy, bilateral salpingo-oophorectomy, omentectomy, and appendectomy (postpartum)	Disease progression; death 24 days later

## Conclusions

Treatment of epithelial ovarian carcinoma should not differ between pregnant and non-pregnant patients, but management of the disease requires the maintenance of a delicate balance between maternal oncological results and fetal well-being. The case presented here represents a rare clinical scenario that shows the aggressive nature of the disease. Information about this disease is limited because of its rarity, so we considered it important to share our experience.
